# Characterisation of the gut microbial community of rhesus macaques in high-altitude environments

**DOI:** 10.1186/s12866-020-01747-1

**Published:** 2020-03-26

**Authors:** Yuhan Wu, Yongfang Yao, Mengmeng Dong, Tianrui Xia, Diyan Li, Meng Xie, Jiayun Wu, Anxiang Wen, Qin Wang, Guangxiang Zhu, Qingyong Ni, Mingwang Zhang, Huailiang Xu

**Affiliations:** 1 College of Life Science, Sichuan Agricultural University, No. 46, Xinkang Road, Yucheng District, Ya’an, Sichuan 625014 People’s Republic of China; 2 College of Animal Science and Technology, Sichuan Agricultural University, Chengdu, 611130 China

**Keywords:** Rhesus macaque, Gut microbial community, 16S rRNA gene, High-altitude environment

## Abstract

**Background:**

The mammal intestinal microbiota is involved in various physiological processes and plays a key role in host environment adaption. However, for non-human primates (NHPs), little is known about their gut microbial community in high-altitude environments and even less about their adaption to such habitats. We characterised the gut microbial community of rhesus macaques from multiple high-altitude environments and compared it to those of low-altitude populations.

**Results:**

We collected faecal samples of rhesus macaques from four high-altitude populations (above 3000 m) and three low-altitude populations (below 500 m). By calculating the alpha diversity index, we found that high-altitude populations exhibited a higher diversity. Statistical analysis of beta diversity indicated significant differences between high- and low-altitude populations. Significant differences were also detected at the phylum and family levels. At the phylum level, the high-altitude gut microbial community was dominated by Firmicutes (63.42%), while at low altitudes, it was dominated by Bacteroidetes (47.4%). At the family level, the high-altitude population was dominated by Ruminococcaceae (36.2%), while the low-altitude one was dominated by Prevotellaceae (39.6%). Some families, such as Christensenellaceae and Rikenellaceae, were consistently higher abundant in all high-altitude populations. We analysed the overlap of operational taxonomic units (OTUs) in high-altitude populations and determined their core OTUs (shared by all four high-altitude populations). However, when compared with the low-altitude core OTUs, only 65% were shared, suggesting a divergence in core OTUs. Function prediction indicated a significant difference in gene copy number of 35 level-2 pathways between high- and low-altitude populations; 29 of them were higher in high altitudes, especially in membrane transport and carbohydrate metabolism.

**Conclusions:**

The gut microbial community of high-altitude rhesus macaques was significantly distinct from that of low-altitude populations in terms of diversity, composition and function. High-altitude populations were dominated by Firmicutes and Ruminococcace, while in low-altitude populations, Bacteroidetes and Prevotellaceae were dominant. The difference in gut microbiota between these two populations may be caused by differences in host diet, environmental temperature and oxygen pressure. These differentiated gut microbial microorganisms may play a critical role in the adaptive evolution of rhesus macaques to high-altitude environments.

## Background

The gastrointestinal tract of animals is habited by a complex microbial community, known collectively as the gut or intestinal microbiota. There is increasing evidence that the gut microbial community of animals is involved in a wide range of processes in the host, including health, grow and development as well as behaviour; it can even affect the nervous system by secreting metabolites [1–11]. These microbes can also assist in energy uptake and metabolism [12, 13]. Previous research has demonstrated that ruminants depend heavily on gut microbiota to digest fibre in the rumen and use products such as short-chain fatty acids (SCFAs) to meet their energy needs [14, 15]. Panda, which do not have a rumen, also rely on their microbiome to degrade hemicellulose and starch in bamboo [16]. The functions of gut microbiota are mainly determined by their composition, which is affected by various factors such as host genetic background, health condition, social behaviour and habitat environment factor [17–24]. Because of the importance of gut microbiota and other microbiome communities, some scientists suggest that the host and its microbiomes are considered as a biological unit subjected to natural selection [25]. When faced with selection pressure, the host or their gut microbiota or both could response to this pressure [26–28].

Understanding how animals adapt to their environments has always been a hot topic in ecology research. High-altitude environments are some of the most extreme environments and challenging habitats for most animals. With increasing elevation, atmospheric pressure decreases drastically, resulting in reduced body oxygen saturation levels (hypoxemia). These changes can suppress aerobic metabolism, which is the main energy production process. Without compensatory adjustments, hypoxemia might develop into critical altitude sickness. High-altitude regions have an alpine climate with low temperatures, increasing the energy requirement of the animals. Against this background, high-quality food, such as fruits and buds, is less abundant in such ecosystems, and animals rely on more low-quality foods such as bark and grass roots, which contain more indigestible carbohydrates, less lipids and fewer proteins. In particular, for endothermic animals, high-altitude environments are highly challenging as they have a higher metabolism rate and typically require more oxygen and energy to sustain their body temperature when compared to ectothermic animals. Despite these extreme conditions, humans and many animals have colonised such areas. Animals adapted to high altitudes might have developed some specific adaptive mechanisms, which have been subject of research for several years. For example, animals living in high-altitude regions have evolved specific strategies in terms of genetics, physiology, morphology and behaviour [29–39]. Recently, some studies have indicated that gut microbiota may assist mammals to adapt to such environments. High-altitude animals, such as Tibetan sheep and yak, possess more SCFA-producing bacteria such as methylotrophic methanogens and *Prevotella* when compared with their low-altitude relatives. Such microbiomes show a stronger fermentation ability and contain more genes for the production of short-chain fatty acids (SCFAs), providing energy to their host across the epithelium, while their relatives in low-altitude regions contain more genes for methane production [40]. The gut microbial community of plateau pika possess more *Prevotella* species, which produce SCFAs, and exhibit a higher alpha diversity and fermentation ability than low-altitude pika [41]. However, such studies have mainly been conducted on high-altitude herbivores, which differ largely from other animals in terms of diet and phylogenetics, impeding general conclusions.

Non-human primates (NHPs) originated from tropical habitats [30] and mostly prefer warm temperatures; they are distributed throughout tropical or subtropical environments including rain forest and savannah, with only a few species radiating into temperate and alpine forest [42]. The gut microbiota of NHP can respond to seasonal environmental changes [26, 27].

The rhesus macaque (*Macaca mulatta*) is a small omnivorous non-human primate. Its anatomy and physiology are similar to those of humans, making it an ideal model in medical and biological research. *Macaca mulatta* is the most widely distributed NHP in the world [43]. In China, it has been listed as a second-class national-level protected animal and is distributed from sea level to the Tibetan Plateau. With a good adaptation to multitude altitude gradients environments, the rhesus macaque is an ideal model for exploring adaptive mechanisms of NHPs to multitude altitudinal gradients. However, studies on the gut microbiota of NHPs living in high-altitude environments are limited. In our previous preliminary study, we found that the gut microbiota of rhesus macaques exhibited significant differences among different altitude gradients [44]. In this study, based on comparative assays and extensive sampling, we want to further explore how the gut microbiota responds to the adaptation process of rhesus macaques to high-altitude environments. This study is the first work describing the gut microbiota of rhesus macaques living in high-altitude environments and enhances our understanding of their adaption mechanisms to high-altitude habitats, providing valuable information for their conservation. Due to their similarity to humans, this study provides a scientific basis for high-altitude medicine.

## Results

### Sequencing profiles

After quality filtering, 7,777,401 16S ribosomal RNA gene sequences were obtained from 73 rhesus macaque faecal samples (106,540 ± 32,452 per sample). The sequences were clustered at 97% sequence identity, and 1842 OTUs were generated (872 ± 127 per sample). The rarefaction curves had already reached a plateau at this sequencing depth **(Fig.****S1**), suggesting that the sequencing depth had met the demand for sub-sequence analysis. Sequences of more than 99.5% were annotated at the phylum level for high-altitude and low-altitude groups. At the family level, the annotation rate for high-altitude groups was 94.5%, while that for low-altitude groups was 98.5%, indicating that high-altitude rhesus macaques have more unknown microbial species in their gut.

### Gut microbiota diversity in high-altitude populations

**Fig. 1:**
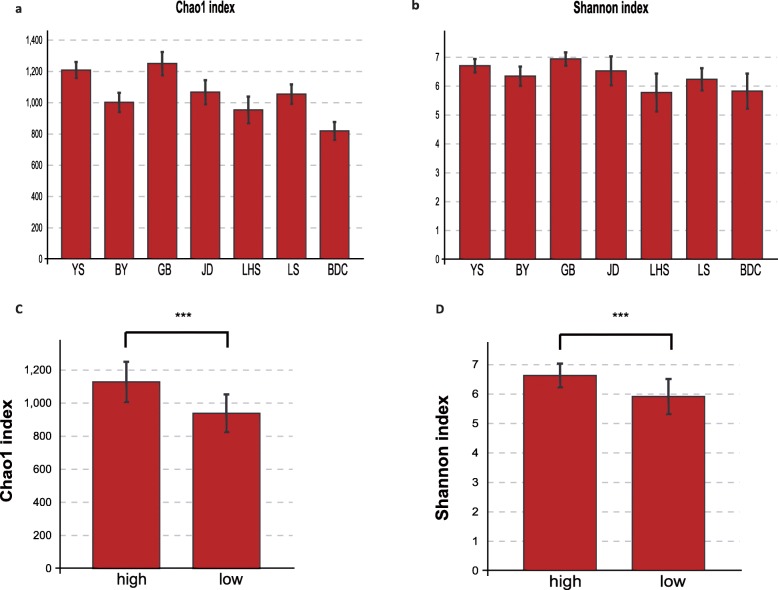
Profile of alpha diversity of seven rhesus macaques populations. By (**a**) Chao1 index, and (**b**) Shannon index. And the comparisons of gut microbiota alpha diversity (**c**) Chao1 index (**d**) Shannon index between high-altitude and low-altitude populations. YS, BY, GB and JD are shorted for four high-altitude populations lived in Qinghai-Tibet Plateau with altitude above 3000 m. LHS, LS and BDC are represented three populations lived in low-altitude areas. High group contains 39 samples collected from 4 high-altitude populations with altitude above 3000 m. Low group include 34 samples from 3 habitat below 500 m. T test was used to test the differences between groups. The significance is indicated by *P < 0.05, **P < 0.01, ***P < 0.001

We calculated the alpha diversity indices to evaluate the gut microbiota diversity in high-altitude populations. First, the Chao1 (1039 ± 150), ACE (1019 ± 145) and sob (872 ± 127) indices were calculated to assess the number of microbial species in high-altitude rhesus macaques **(Table****S1****)**. Specifically, the high-altitude groups GB and YS showed high Chao1 index values of 1247 and 1206, respectively, while those of JD and BY were lower (1064 and 999, respectively) **(**Fig. 1a**)**. In addition, even for rhesus macaques living in similar high-altitude environments, the Chao1 index varied within high-altitude groups (two-tailed t-test *P* < 0.05), except between GB and YS (two-tailed t-test *P* = 0.17). In low-altitude groups, the Chao1 index values were 951, 1052 and 817 for LHS, LS and BDC, respectively. Specifically, when compared with low-altitude populations, high-altitude groups exhibited a significantly higher alpha diversity based on the Chao1 index **(**Fig. 1c two-tailed t-test *P* < 0.0001**)**. To evaluate the evenness of these microbiota, we calculated the Shannon (6.2831 ± 0.6091) and Simpson (1-D = 0.9519 ± 0.03464) indices **(**Fig. 1b; **Table****S1****)**. The order of the Shannon index was similar to the result of the Chao1 index in high-altitude groups. In low-altitude groups, LHS had a significantly higher Chao1 index than BDC (two-tailed t-test *P* < 0.0001). Regarding the Shannon index, BDC had a slightly higher alpha diversity than LHS, although this difference was not significant (*P* > 0.05). Comparing high-altitude groups with low-altitude ones, the Shannon index was higher in the first group (Fig. 1d two-tailed t-test for Shannon *p* < 0.001). Other alpha diversity indices such as ACE, sob and Simpson indices, also confirmed that the high-altitude populations had a relatively high alpha diversity. Our results suggest that although alpha diversity varied among the four high-altitude populations, high-altitude populations generally exhibited a higher alpha diversity.

**Fig. 2:**
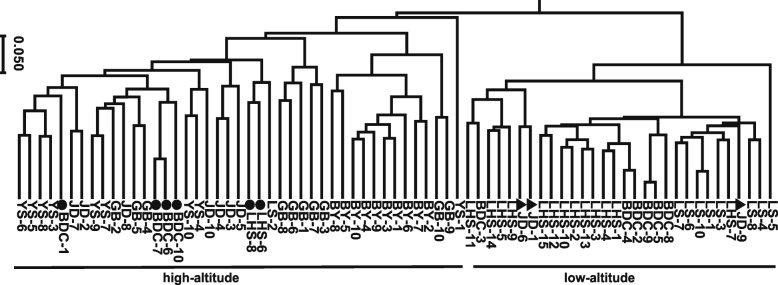
UPGMA cluster plot based on weighted Unifrac distance. It displays that high-altitude groups (BY, YS, GB and JD) and low-alitude groups (LS, LHS and BDC) are separated into two clade indicating they have distinctive gut microbial community. Triangle and circle are used to indicate samples who strays from high-altitude and low-altitude groups respectively

We determined the beta diversity index to analyse the gut microbial community structure for high-altitude and low-altitude rhesus macaques. To take abundance changes and phylogenetic relationships into account, we selected unweighted Unifrac distance and weighted Unifrac distance as indicators of beta diversity. Based on weighted and unweighted Unifrac distances, we conducted UPGMA cluster analysis to visualise the results for beta diversity. Based on the unweighted Unifrac UPGMA clustering tree, all samples were clearly clustered by regional distribution, while the low-altitude population could not be differentiated from the high-altitude one **(Fig.****S2****)**. On the contrary, in the weighted Unifrac UPGMA clustering tree, all groups and most samples were clustered by altitude, although some samples were deviated from their altitude and clustered into opposite clades **(**Fig. 2**)**.

**Fig. 3:**
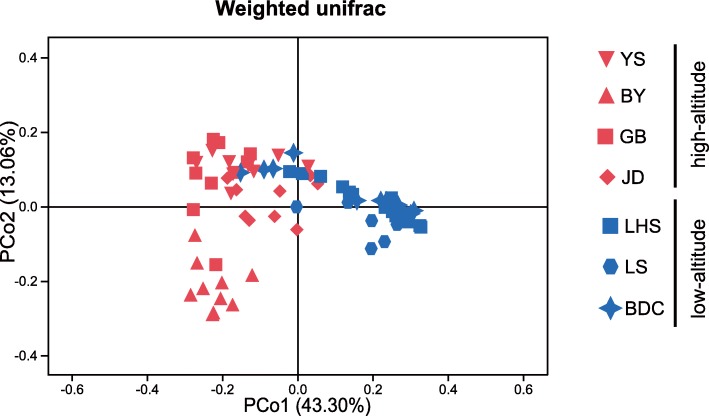
The principal coordinate plot based on Weighted Unifrac distances. Red symbols are marked for high while blue symbols for low. Specially, red triangles are marked for high-altitude group BY which is separated with other high-altitude groups with a distance

Subsequently, we performed PCoA to directly visualise the relationship of beta diversity distance between high-altitude and low-altitude populations. In the Weighted unifrac PCoA plot, samples from the high-altitude populations GB, YS and JD exhibited a closer relationship, while BY was distributed below the X-axis and separated from the other high-altitude samples **(**Fig. 3**)**. The high-altitude populations were concentrated on the left of the Y-axis and separated from the low-altitude ones. Based on our results, Pcoa1 accounted for 43.30% of the total difference, while Pcoa2 accounted for 13.06%. This leads us to infer that the gut microbial community structure of high-altitude populations differs from that of low-altitude ones. To further validate these differences, we conducted an analysis of similarity (ANOSIM) on Bray and weighted Unifrac distance results. The ANOSIM results proved that there were significant differences between low-altitude and high-altitude populations (Bray distance r = 0.5490, *P* < 0.001; weighted Unifrac distance r = 0.5914, *P* < 0.001). We also performed a permutational multivariate analysis of variance (PERMANOVA) on Bray and weighted Unifrac distance results; the PERMANOVA results coincided with those of the ANOSIM (Bray distance r = 0.1760, *P* < 0.001; weighted Unifrac distance r = 0.2901, *P* < 0.001).

Our data strongly suggest that the gut microbiota of high-altitude populations has a high alpha diversity. When including abundance information (weighted), the low-altitude population could clearly be distinguished from the high-altitude population.

### Microbial community structure in high-altitude populations

**Fig. 4:**
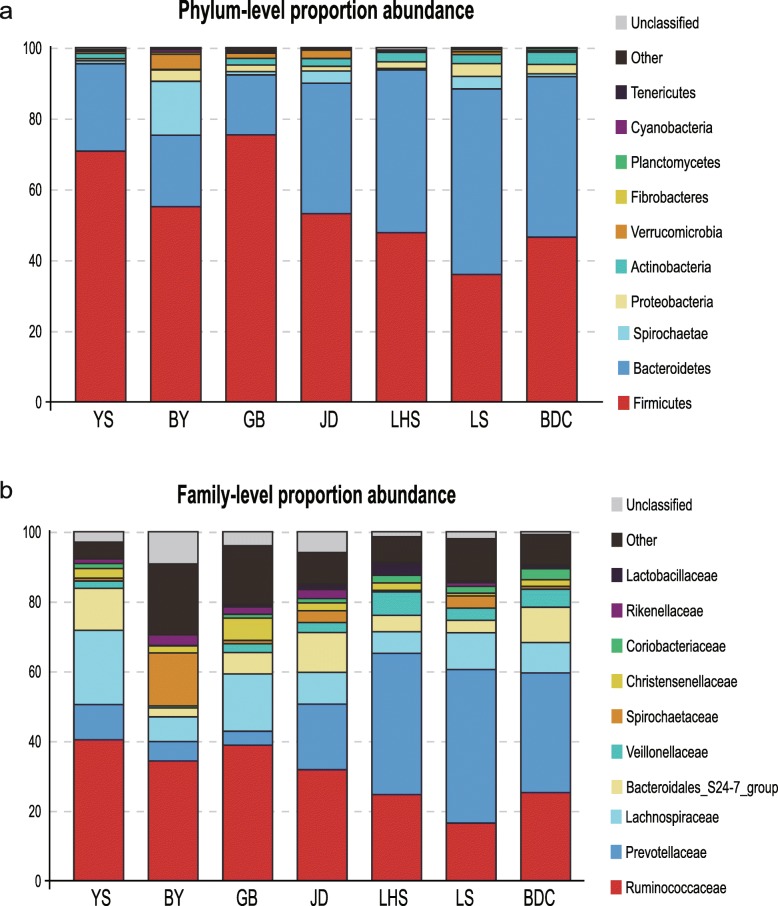
The compositions of rhesus macaques gut microbiota. At (**a**) phylum level and (**b**) family level. Abundance out of top 10 are be classified as other in this plot. High-altitude populations include YS, BY, GB and JD. Low-altitude groups consitst of LHS, LS and BDC.

The predominant phyla of high-altitude rhesus monkey populations were Firmicutes (63.42 ± 14.51%) and Bacteroidetes (24.65 ± 12.11%; Fig. 4a;). Firmicutes were highest in the GB group, accounting for 75.39%, and only slightly lower in the YS group (70.79%). In the BY, they accounted for 55.10% and in the JD group for 53.25%. These numbers were lower in low-altitude groups, with 47.75, 35.99 and 46.51% for LHS, LS, and BDC, respectively. The t-test confirmed that high-altitude populations had more Firmicutes than low-altitude groups (*P* < 0.001). We observed a negative correlation between Firmicutes and Bacteroidetes, which means the proportion of Bacteroidetes in GB (16.91%) was lower than those in the other high-altitude populations such as BY (20.20%) and YS (24.68%). Notably, the Bacteroidetes of JD were significantly more abundant than in the other high-altitude groups, but still lower than in the low-altitude groups. In general, Firmicutes and Bacteroidetes accounted for about 90% of the rhesus macaques gut microbiome. The subordinate phyla were Spirochaetae (5.19 ± 7.41%), Verrucomicrobia (2.18 ± 1.40%), Proteobacteria (1.76 ± 0.94%) and Actinobacteria (1.42 ± 0.78%), while the other phyla accounted for less than 1%. Specifically, when compared with the low-altitude group, high-altitude populations contained more Firmicutes (two tailed t-test *P* < 10^− 9^) but less Bacteroidetes (two tailed t-test *P* < 10^− 5^). Thus, the ratio of Firmicutes to Bacteroidetes in high-altitude populations was more than three times as high as that of low-altitude populations.

**Fig. 5:**
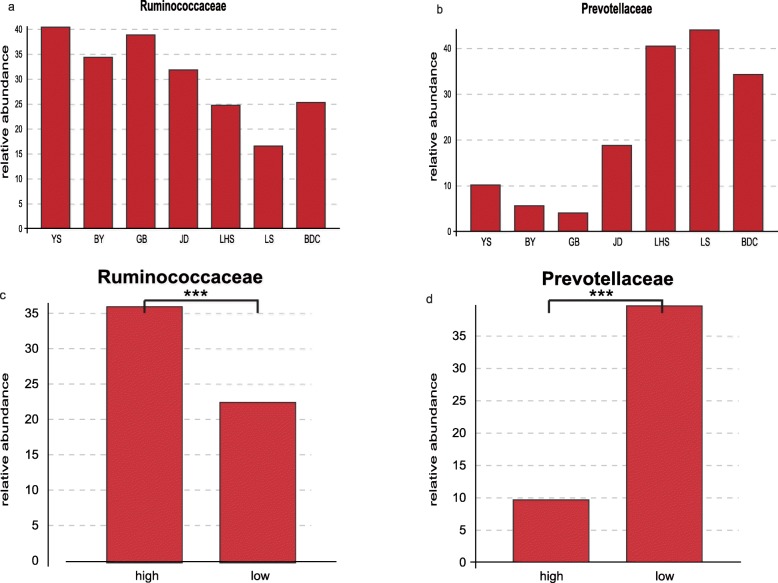
The high abundance family level of rhesus macaques gut microbiota. (**a**) and (**b**) and the comparison of high abundance microbiota at family-level between high-altitude populations and low-altitude populations(**c**) and (**d**). YS, BY, GB and JD are shorted for four high-altitude populations lived in Qinghai-Tibet Plateau with altitude above 3000 m. LHS, LS and BDC are represented three populations lived in low-altitude areas. T test was used to test the differences between groups. The significance is indicated by *P < 0.05, **P < 0.01, ***P < 0.001

**Fig. 6:**
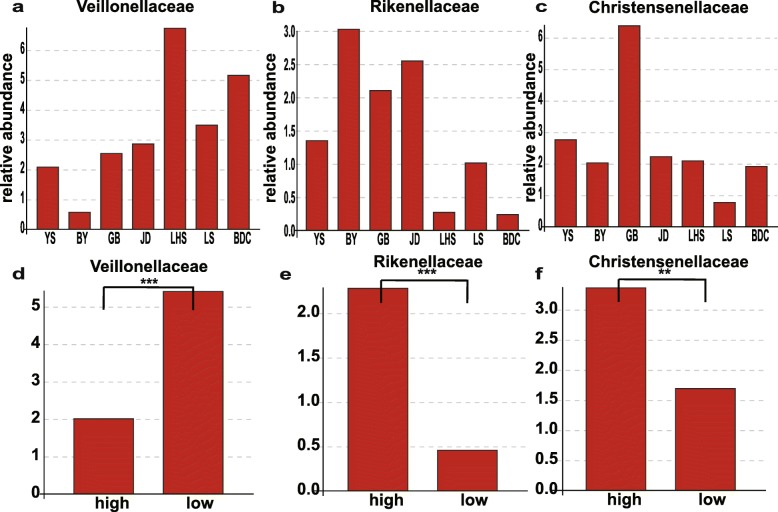
Compositions and comparison of low abundance family between two altitudes. YS, BY, GB and JD are shorted for four high-altitude populations lived in Qinghai-Tibet Plateau with altitude above 3000 m. LHS, LS and BDC are represented three populations lived in low-altitude areas. The significance is indicated by *P < 0.05, **P < 0.01, ***P < 0.001

**Fig. 7:**
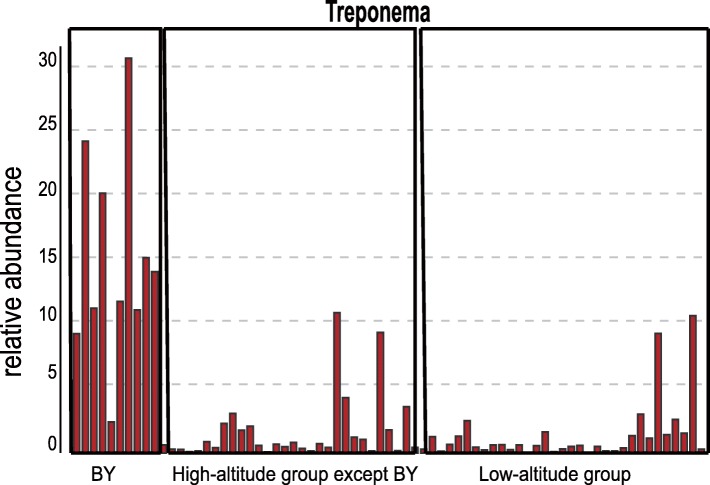
The abundance of Treponema in all samples. BY group other high-altitude groups and low-altitude groups has been marked with box. T-test indicate Treponema is significant higher in By group than Rhesus macaques other populations (*P* < 0.05 for all populations significance symbol was not shown in the picture)

At the family level, the annotation rate in high-altitude groups was about 94.5%, while that in low-altitude groups was about 98.5%. Ruminococcaceae (36.23 ± 9.74%), Lachnospiraceae (13.27 ± 7.66%) and Prevotellaceae (9. 60 ± 9.08%) were the dominant families in high-altitude populations; other represented family were Bacteroidales_S24-7_group (7.91 ± 7.26%), Spirochaetaceae (5.20 ± 7.41%), Christensenellaceae, Rikenellaceae and Veillonellaceae. These family accounted for more than 80% sequences in total (Fig. 4b). Particularly, the abundance of Prevotellaceae was highest in JD, while that of Ruminococcaceae was lowest. To further explore the distinguishing feature in species composition in high-altitude populations, the t-test was used at the family level. The abundances of the gut bacterial communities significantly differed among samples from different altitudes. Ruminococcaceae (two tailed t-test *P* < 0.01) were highest in the high-altitude population, while Prevotellaceae were highest in the low-altitude population (Fig. 5) In addition, Ruminococcaceae and Prevotellaceae were the largest families in the high-altitude and low-altitude populations, respectively. We also observed differences in the abundances of the families Veillonellaceae, Rikenellaceae and Christensenellaceae (Fig. 6**;** two-tailed t-test *P* < 0.005); such tendencies were found for all four high-altitude populations. We therefore suggest that high-altitude rhesus macaques have a similar gut microbiome. Notably, although Spirochaetae was a subdominant phylum, it was significantly higher abundant in the BY population when compared with other high-altitude groups. Removing the BY group from the analysis reduced the Spirochaeta level in high-altitude populations from 5.19 to 1.74%. These Spirochaetae in the BY group can be further assigned to the genus *Treponema* (Fig. 7).

**Fig. 8:**
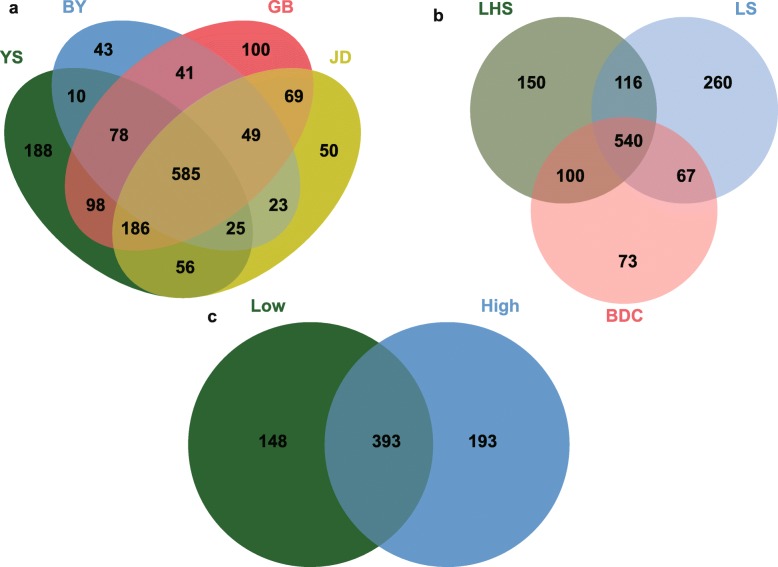
The venn plot of all high and low altitude groups. (**a**) core OTUs of high altitude groups and (**b**) low-altitude groups, and (**c**) core OTUs between high-altitude and low-altitude groups. Numbers in plots are marked for how many OTUs are in this part. YS, BY, GB and JD are shorted for four high-altitude populations lived in Qinghai-Tibet Plateau with altitude above 3000 m. LHS, LS and BDC are represented three populations lived in low-altitude areas

Based on the OTU data, we generated a Venn diagram to explore the common OTUs among the populations. To improve the reliability of our data and to reflect the gut microbiota community of each group, we filtered OTUs with abundance levels below 10 in a single sample, and 1863 OTUs remained. Furthermore, 585 OTUs were shared by all four high-altitude populations and were the core OTUs of high-altitude rhesus macaques. The core OTUs of three low-altitude groups consisted of 540 OTUs. When calculated in terms of abundance, core OTUs accounted for about 90%. When we compared the core OTUs between high-altitude and low-altitude groups, we found that only 393 OTUs were shared, which means that high-altitude groups had 193 unique OTUs which do not occur in low-altitude groups (Fig. 8). Most of these unique OTUs (88 of 193) belonged to the family Ruminococcaceae, while 31 OTUs belonged to the Clostridiales_vadinBB60_group, 16 to Lachnospiraceae and 21 could not be assigned to one family. These unique OTUs accounted for 4.36% in high-altitude rhesus macaques. Although their abundance might be relatively small, they consistently occurred in all high-altitude populations, indicating their importance at high altitudes.

### Functional profiling of microbiota in high-altitude populations

To evaluate metabolic function differences between gut microbiota communities associated with high altitudes, we used Phylogenetic Investigation of Communities by Reconstruction of Unobserved States (PICRUSt). Microbial community function was assigned based on the Kyoto Encyclopedia of Genes and Genomes (KEGG), including 35 level 2 and 221 level 3 ortholog groups. The gene copy numbers of the gut microbiome were significant higher in high-altitude rhesus macaques than in the low-altitude group (t-test *P* < 0.0001). Thus, there were a large number of differences pathway between the two populations, including 30 level 2 and 180 level 3 ortholog groups (t-test *P* < 0.05). Strikingly, almost all different pathways were more highly expressed in the high-altitude population. Functions enriched in high-altitude populations were involved in a wide range of processes, for instance, membrane transport, carbohydrate metabolism, amino acid metabolism, replication and repair, translation, energy metabolism (**Fig.****S3**).

Our data indicate that pervasive biological processes were elevated in the intestinal microbes of high-altitude rhesus macaques when compared with low-altitude populations, which was mainly the result of the abundance difference of the common OTUs.

## Discussion

### Characteristics of high-altitude rhesus macaques in terms of diversity

High-altitude populations exhibited a high multivariate alpha index, similar to high-altitude pika [41]. There is increasing evidence that the gut microbiome harbours 130 glycoside hydrolase, 22 polysaccharidelyase and 16 carbohydrate esterase families [45]. A high alpha diversity reflects a higher number of microbial species which may contain more cellulose-associated gene. Gene diversity provides diverse parallel pathways, resulting in a high microbiome flexibility and a high capacity to use different cellulose sources [41]. Thus high-altitude rhesus macaque populations with high diversity might has a higher ability to utilize high-fiber food to help them meet energy requirement in cold high-altitude habitats. The high-altitude groups BY and JD showed a lower alpha diversity than other high-altitude populations. Interestingly, both groups live close to the Yangtse River, which leads us to infer that the Yangtse River provides a relatively luxuriant vegetation. Thus, rhesus macaques in this environment may have more available food resource and do not heavily rely on microbiota diversity to assimilate food.

On the other hand, a recent study has revealed that our bodies have the ability to control microbial communities by restricting nitrogen access to starve gut bacteria or by secreting nitrogen via the intestinal cells to feed bacteria [46]. Compared to high-altitude populations, low-altitude population consume more high-nitrogen food, for example fruit, which might weaken the control from the host. In our data, OUT abundance was highest for low-altitude areas, with 12,724, 5303, 4381, 3167 and 2439, respectively. In high-altitude areas, the values were 5874, 4469, 4129, 3626 and 3103. In this sense, some bacteria in low-altitude populations might propagate excessively and finally lead to both low OTUs and low evenness, which is the premonition of imbalance. A similar phenomenon has been observed in humans consuming a Western diet with large amounts of fat and protein [47].

The gut microbiota composition is shaped by various factors such as genetic background, diet, environmental differences and social behaviour [20, 25, 48, 49]. In the same population, animals have a similar genetic background and diet, and therefore the gut microbiota composition of rhesus macaques is expected to be more similar for animals of the same group. The results shown in the unweighted Unifrac UPGMA cluster dendrogram show that the samples are clustered as geographical populations (**Fig.****S2**). However high-altitude populations cannot be distinguished from low-altitude populations in this unweighted dendrogram. Interestingly, in the weighted dendrogram, high-altitude populations are separated from low-altitude populations, but individuals are not clustered as geographical populations. These data suggest that high-altitude environments may shape the gut microbiota composition by changing their microbiota abundance, and these convergent changes are greater than the differences caused by the genetic background, geographical position and social relationships.

Composition difference between high-altitude and low-altitude rhesus macaque populations.

The gut microbial community of high-altitude rhesus macaques was dominated by Firmicutes and Bacteroidetes, similar to the low-altitude population. However, in high-altitude populations, the ratio of Firmicutes/Bacteroidetes was higher than in the low-altitude group. Specifically, the values for the four high-altitude populations were 2.8, 1.4, 4.45 and 2.7, while for the low-altitude populations, the values were 0.69, 1.0 and 1.0, with a significant difference (*p* < 0.001). These results are in agreement with a previous study on the Tibetan Plateau [50]. Firmicutes are considered to encode energy metabolism-related enzymes and can produce various digestive enzymes to decompose various substances, while Bacteroidetes degrade carbohydrates and proteins [51]. Similar results have been observed in cold-exposed mice and were associated with increased non-shivering thermogenesis and energy harvest, indicating that high-altitude populations have high energy harvest and consumption [52].

The high F/B values contributed to the increased abundance of the family Ruminococcaceae (Firmicutes) and the decreased abundance of the family Prevotellaceae (Bacteroidetes). Ruminococcaceae were enriched in high-altitude populations, while Prevotellaceae were enriched in low-altitude populations. Based on a previous study, Ruminococcaceae play a role in cellulose degradation [45]. Via microbial fermentation, cellulose can be transformed into short-chain fatty acids (SCFA), which are an important energy source for epithelium and can provide about 10% of the energy for humans [53, 54]. In black howler monkeys, Ruminococcaceae abundance increases during the energy-scarce period and appears to compensate for the reduced energy intake [26]. In high-altitude habitats, animals subjected to hypoxia and cold environments require more energy to maintain their metabolic balance, but available food sources are limited and mainly consist of lignin and cellulose (as in roots and bark), which cannot be used by the hosts [26]. In this sense, an increased abundance in Ruminococcaceae leads an an increased efficiency in energy intake and supports rhesus macaques living in cold, high-altitude environments. *Prevotella* species can efficiently use carbohydrates [55], and previous studies have found a positive correlation between Prevotellaceae and fruit consumption in NHPs such as western lowland gorillas and Verreaux’s sifakas [54, 56]. These findings are in agreement with our observation that high-altitude habitats with a plateau climate have a limited fruit production. However, according to a different study, high-altitude ruminants possess more *Prevotella* species than their relatives in low-altitude habitats, and this kind of microbiome produces more energy though fermentation [40]. However, there are also differences in the phylogenetic relationship, physiology and food habits between ruminants and NHPs, which are all factors influencing the gut microbial community [57]. This discrepancy implies that various species may have an altered gut microbiota composition as an adaptation to high-altitude environments.

Our previous work based on elevational gradients found that the abundance of Christensenellaceae was significantly higher in Tibet (3427 m) than in other geographical populations (5, 158, 1161, 1629 2895 m) [44]. However, our study has several limitations, of which the main one is that only one high-altitude site with six samples was included, which may not represent the universal features of the microbiota composition in high-altitude environments. In this study, the abundance values of Christensenellaceae in the high-altitude populations BY, JD, GB and YS were 2.0, 2.2, 6.4 and 2.6%, respectively. However, the values in low-altitude populations were 1.9 2.1 and 0.77% for BDC, LHS and LS, respectively, which were significantly lower when compared to those of all high-altitude populations (t test *p* < 0.01). Although we removed the highest group GB (6.4%) in the high-altitude population, this discrepancy still existed. Therefore, we infer that Christensenellaceae has a higher abundance in all high-altitude rhesus macaque populations and may play a critical role in the adaptation to such environments. Previous research found that Christensenellaceae is significantly correlated with lean body shape (body mass index (BMI) < 25), and the abundances of Christensenellaceae was highly associated with lower triglyceride levels [19, 58, 59]. However, these studies did not show the specific mechanism by how Christensenellaceae interacts with host physiology and how it reduces weight and total adiposity gain. In our study, high-altitude rhesus macaques living a cold environment have a high energy requirement, which is proved by increased abundance of Firmicutes. Therefore, Christensenellaceae species are less likely to reduce weight by suppressing energy harvest. We speculate that the underlying mechanism is based on Christensenellaceae stimulating host metabolic rate and increasing energy consumption. This high metabolic rate could help rhesus macaques maintain their body temperature during the cold winter. This hypothesis is partial implied by another study that observed that the relative abundance of Christensenellaceae was positively correlated with food intake, body mass change and athletic ability [60].

Specially, we every population contained *Treponema* (Spirochaetae) at the proportion of 1–3%, except the BY group, in which this genus accounted for up to 15%. Usually, *Treponema* is known for *T. pallidum*, the cause of syphilis and yaws. This genus also includes proficient cellulose and xylan hydrolysers and is pervasive in NHPs, but rare in industrialised human populations [61, 62]. In a study on western lowland gorillas, *Treponema* was the only genus associated with low fruit yield [56]. A high abundance of *Treponema* could be an adaptation to diets rich in fibre, and possibly, Treponema facilitates the extraction of nutrients from fibrous foods by degrading fibre. The high *Treponema* content in the BY group may imply less desirable food and high fibre intake, but the specific factor needs further investigation of surrounding areas.

While widespread in NHPs, high amounts of *Treponema* are only found in a few rural communities, such as the Hadza population, which has a diet rich in fibre [62]. Fibre degraders can help release nutrients from fibre-rich foods. On the other hand, such foods can promote the growth of these species. A western diet, which generally contains high amounts of fat and low amounts of cellulose, may lead to the exhaustion of fibre degraders, playing an important role in evolutionary history. In this sense, studies focusing on the gut microbiota of wild NHPs may provide an excellent view on microbiome-associated human diseases.

In the Venn diagram, all high-altitude rhesus macaque groups shared 585 OTUs (core OTUs), while all low-altitude populations shared 540 OTUs. These core OTUs are shared by all populations living at the same altitude, but with a high geographical distance, and constitute most of the microbiome of an individual, which means they may be critically important to the essential function of rhesus macaques. However, only 65% of the core OTUs were shared by high-altitude and low-altitude populations. Although those un-shared core OTUs are less numerous, they occur consistently in all high-altitude populations, indicating that they play an important role in the adaptation to high-altitude environments.

### Pervasive enrichment of gut microbiota function in high-altitude rhesus macaques

All mammals have a symbiotic relationship with their microbial community, which contains 10^13^ bacterial cells and more than 3 million genes, enabling the community to carry out multiple functions [63, 64]. Unlike the host genome, the microbiome can change the composition of the microbial community or evolve rapidly in terms of individual microbial genes, resulting in modified transcriptomic, proteomic and metabolic profiles [65, 66]. Recent research has revealed that gut microbiota of NHPs vary among seasons [26, 27]. The dynamic fluctuations in the microbiota community are associated with tremendous changes in gene copy number and appear to facilitate host adaption to their environment. In this study, we exerted PICRUSt to infer the function of the rhesus macaque gut microbial community. We suppose that genes involved in metabolism may be enriched in high-altitude populations.

Spectacularly, almost all gene copy numbers were higher in high-altitude populations than in low-altitude ones. In level two, the top five pathways at both altitudes were membrane transport, carbohydrate metabolism, amino acid metabolism, translation, replication and repair. All pathways were significantly higher expressed in high-altitude populations. ATP-binding cassette (ABC) transporters, the most highly expressed pathway in the membrane transport, is directly involved in ATP generation. Genes enriched in carbohydrate metabolism, translation and amino acid metabolism suggest high metabolism capacity of the gut microbial community. Low oxygen levels and high ultraviolet radiation may cause DNA and protein impairment in high-altitude environments, and genes related to replication and repair are conducive to reduce the damage of biological molecules. Thus, these pathways may help rhesus macaques adapt to high-altitude environments. In terms of abundance, only the pathways glycan biosynthesis and metabolism were significantly higher expressed in low-altitude populations (Wilcoxon’s rank sum test *p* < 0.05) and are concentrated in lipopolysaccharide biosynthesis. Lipopolysaccharides are the major components of the outer membrane of Gram-negative bacteria and also typical endotoxins [67]. This is in agreement with our hypothesis that excessive nutrition in low-altitude populations results in the reproduction of specific bacteria.

## Conclusions

High-altitude rhesus macaques possess a distinct gut microbial community when compared with low-altitude populations. Changes in the gut microbiota were mainly caused by the high-altitude environment, which is characterised by the production of high-fibre food and cold temperatures, leading to hypoxia.

## Methods

### Ethics statement and faecal sample collection

Prior to sampling, the experiment was approved by the Institutional Animal Care and Use Committee of the Sichuan Agricultural University (permit number SKY-S20171007). All field works were granted permission by the Administration for Wild Animal and Plant Protection and Nature Reserves and the Department of Forestry for the Tibet provincial region, as well as Hainan, Guangxi, Qinghai, Chongqing and Sichuan provinces. All faecal samples (*n* = 75) were collected at natural habitats with little disturbance by humans and livestock. Samples were collected with sterile gloves immediately after each wild rhesus macaque had defecated and were kept cool in a thermos with ice pack. The samples used for bacterial DNA preparation were taken from the inside of the faeces under sterile conditions. Subsequently, the samples were transported to the laboratory on dry ice and stored at − 80 °C before DNA extraction.

**Fig. 9:**
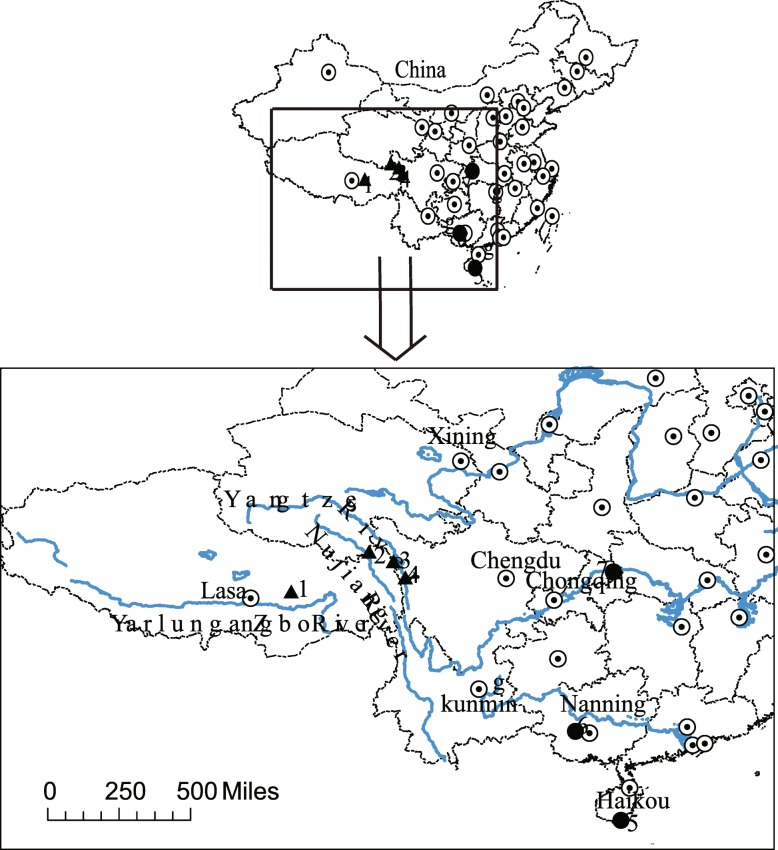
Sample collection map. The base county-level vector map was download and extracted freely from the GADM database (www.gadm.org), version 2.8, and the collection sites are inserted by longitudinal and latitudinal coordinate. Rivers are showed in blue lines and high-altitude populations are marked with triangle while low-altitude site are marked with circle. Number 1 to 4 were marked for our high-altitude populations 1-GongBujiangda about 3200 m 2-YuShu 4080 m 3-JiangDa 3246 m 4-BaiYu 4091 m. These sites were shorted as GB, YS, JD and BY respectively. Number 5, 6, and 7 were marked for low-altitude populations. 5-LingShui 100 m 6-LongHuShan 150 m 7-BaiDiCheng 150 m. These sites were shorted as LS, LHS and BDC

This study included seven rhesus macaque populations; groups in high-altitude habitats and three in low-altitude habitats **(**Fig. 9**,** Table 1**)**. The low-altitude faecal samples were collected at LingShui, LongHuShan and BaiDiCheng (LS, LHS and BDC, respectively). All low-altitude samples were collected at habitats with an average elevation below 500 m. The site LS (in Hainan province) has a tropical monsoon climate with high rainfall and high temperatures, producing a considerable amount of high-quality food (mainly mangoes and coconuts) throughout the year, facilitating rhesus macaque colonisation. The sites LHS and BDC have a subtropical monsoon climate with temperatures lower than in LS, harbouring subtropical evergreen forests. The high-altitude habitats at the Tibetan Plateau, with a altitude above 3000 m, included JiangDa, BaiYu, YuShu and GongBujiangda (JD, BY, YS and GB, respectively). The climate in these habitats was significantly colder with less rainfall. The JD population was relatively close to the BY one (crow-fly distance of 70 km), but separated by the Yangtze River. This allowed us to determine whether the river could be a potential geographical barrier for the gut microbial community of rhesus macaques.

**Table 1:** Sample collection information

Sample collection information									
location	abbreviate	altitude	climate	annual mean rainfall	annual average temperature	mean temperature in January	sample number	vegetation	groups
BaiYu	BY	4091 m	alpine subarctic climate	600 mm	6.3 °C	−4.4 °C	10	coniferous forest and alpine meadows	High-altitude groups (*n* = 40)
JiangDa	JD	3246 m	alpine subarctic climate	550 mm	4.5 °C	−5 °C	10	coniferous forest and alpine meadows	
GongBuJiangDa	GB	3200 m	alpine subarctic climate	640 mm	8.3 °C	−0.4 °C	10	coniferous forest and alpine meadows	
YuShu	YS	4080 m	alpine subarctic climate	480 mm	5.9 °C	−4.5 °C	10	coniferous forest and alpine meadows	
LingShui	LS	100 m	tropical monsoon climate	1700 mm	25.2 °C	17.7 °C	10	a deserted plantation with mango and coconut	Low-altitude groups (*n* = 35)
LongHuShan	LHS	150 m	subtropical humid monsoon	1300 mm	21.8 °C	13.2 °C	15	subtropical evergreen forest	
BaiDiCheng	BDC	150 m	subtropical humid monsoon	1100 mm	16.4 °C	8.6 °C	10	subtropical evergreen forest	

### DNA extraction and sequencing

The DNA was extracted from faecal samples using a TIANamp Stool DNA kit (Tiangen, Beijing, China), following the manufacturer’s instructions. The integrity of the extracted genomic DNA was verified by 1.0% agarose gel electrophoresis. The V3-V4 regions of the bacterial 16S rRNA gene (from 341 to 806) were amplified from extracted DNA using the barcoded primers 341 F (5′- CCTACGGGNGGCWGCAG − 3′) and 806 R (5′-GGACTACNVGGGTATCTAAT-3′). The PCR was performed in a 50-μL reaction system containing 1.5 μL of each primer, 100 ng template DNA, 5 μL 10 × KOD Buffer, 5 μL 2.5 mM dNTPs and 1 μL KOD polymerase. The PCR conditions consisted of a denaturation step at 95 °C for 2 min, and amplification was carried out with 27 cycles at a melting temperature of 98 °C for 10 s, an annealing temperature of 62 °C for 30 s and an extension temperature of 68 °C for 30 s. The final extension step was performed at 68 °C for 10 min. The barcoded PCR products were purified using a DNA gel extraction kit (Axygen, China) and quantified using QuantiFluorTM real-time PCR. Subsequently, next-generation sequencing was performed by Illumina Hiseq 2500 PE250, which was conducted by Genedenovo Inc. (Guangzhou, China).

### Data analysis

Low-quality sequences that contained more than 10% of the ambiguous base (N) were removed, and sequences with less than 60% of the high-quality base (quality score above 20) were also removed. Subsequently, tags were merged using the FLASH program (version 1.2.11) with default parameters. Low-quality contigs were removed using Qiime (V1.9.1) [68]. After removing chimera, the software MOTHUR was used to remove the redundant tags to obtain unique tags [69, 70]. The obtained unique tags were then used to calculate the abundance. The demultiplexed reads were clustered at 97% sequence identity into operational taxonomic units (OTUs) using the UPARSE pipeline [71]. To avoid low abundance bacterial contamination from reagents [72], OTUs with low abundance were removed. and OTUs were classified using the RDP classifier, the Silva version 123 and the Greengenes version gg_13_5 reference set with an 80% confidence threshold [73–75]. After annotation, two samples (YS-2 LS-9) were abnormally high Proteobacteria and Actinobacteria, which may reflect the dysbiosis in gut microbiota [76]. These samples were therefore removed from the subsequent analyses.

The alpha diversity indices Shannon, Simpson, Chao1, ACE, as well as rarefaction curves, were calculated using Qiime. The beta diversity metrics, the Bray distance and weighted UniFrac distance matrices were calculated and visualised in the R statistical software. Statistics of Welch’s t-test, the Wilcoxon rank sum test, ADONIS and ANOSIM were also calculated using R. The functions of gut bacterial communities were predicted using phylogenetic investigation of communities by reconstruction of unobserved states (PICRUSt version 1.1.0) [77].

## Supplementary information

**Additional file 1: Figure S1.** The rarefaction curves of all samples. Which are marked with different colour on the right.

**Additional file 2:Figure S2.** UPGMA cluster plot based on unweighted Unifrac distance. It display that high-altitude groups (BY, YS, GB and JD) and low-alitude groups (LS, LHS and BDC) can’t be separated into two clade.

**Additional file 3:Figure S3.** The comparison of gene copy numbers of gut microbiota between high-altitude and low-altitude Rhesus macaques populations. T test was used to test the differences between groups. The significance is indicated by *P < 0.05, **P < 0.01, ***P < 0.001

**Additional file 4.**

## Data Availability

The datasets used and/or analysed during the current study are available from the corresponding author on reasonable request.

## Abbreviations

16S rRNA: 16S ribosomal RNA
ANOSIM: Analysis of similarity
Chao1: Chao1 estimator
KEGG: Kyoto Encyclopedia of Genes and Genomes
NHPs: Non-human primate
OTU: Operational taxonomic unit
PERMANOVA: Permutational multivariate analysis of variance
PICRUSt: Phylogenetic Investigation of Communities by Reconstruction of Unobserved States
SCFAs: Short-chain fatty acids
UPGMA: Unweighted pair-group method with arithmetic means
